# Rethinking Autism Intervention Science: A Dynamic Perspective

**DOI:** 10.3389/fpsyt.2022.827406

**Published:** 2022-02-25

**Authors:** Yun-Ju Chen, Eric Duku, Stelios Georgiades

**Affiliations:** Department of Psychiatry and Behavioural Neurosciences, McMaster University, Hamilton, ON, Canada

**Keywords:** autism (ASD), intervention outcome, developmental trajectories, time-varying (TV), longitudinal, prediction

## Abstract

Recent advances in longitudinal methodologies for observational studies have contributed to a better understanding of Autism as a neurodevelopmental condition characterized by within-person and between-person variability over time across behavioral domains. However, this finer-grained approach to the study of developmental variability has yet to be applied to Autism intervention science. The widely adopted experimental designs in the field—randomized control trials and quasi-experimental designs—hold value for inferring treatment effects; at the same time, they are limited in elucidating *what* works for *whom, why*, and *when*, given the idiosyncrasies of neurodevelopmental disorders where predictors and outcomes are often dynamic in nature. This perspective paper aims to serve as a primer for Autism intervention scientists to rethink the way we approach predictors of treatment response and treatment-related change using a dynamic lens. We discuss several empirical gaps, and potential methodological challenges and opportunities pertaining to: (1) capturing finer-grained treatment effects in specific behavioral domains as indexed by micro-level within-person changes during and beyond intervention; and (2) examining and modeling dynamic prediction of treatment response. Addressing these issues can contribute to enhanced study designs and methodologies that generate evidence to inform the development of more personalized interventions and stepped care approaches for individuals on the heterogeneous spectrum of Autism with changing needs across development.

## Introduction

Over the past decade, the field of Autism intervention science has made significant advances that led to promising evidence on improving the developmental outcomes of individuals with Autism (1). However, methodological concerns such as small sample size, detection bias related to limited informant types and objective outcome measures, and restricted trial contexts, continue to limit the replicability and generalizability of these findings (1–3). Recent meta-analytic studies revealed empirical gaps in the prediction of differential treatment response and mechanisms through which treatments work, potentially due to limited statistical power and discrepancies in designs and reporting practices across studies (1, 4).

Certain conceptual limitations in manipulating and evaluating treatment-related change could also be a barrier to advancing personalized care in Autism. Specifically, Autism intervention science has historically relied on traditional randomized control trials (RCTs) and quasi-experimental methodologies that often do not account for the heterogeneous and dynamic nature of Autism. While being useful in yielding causal inferences of treatment effects, RCTs evaluating Autism interventions use the process of randomization to “control” statistically for the possible influence of static “confounding” factors at baseline not under direct experimental control. Also, treatment response in RCTs is often determined by comparing group (experimental vs. control) differences or subtracting placebo response from the overall response, thus being limited in assessing individual-level treatment response (5). This is particularly relevant to Autism, as we know from observational longitudinal studies that variable developmental trajectories can be identified among autistic individuals over the life span in several behavioral domains that are often the targets of Autism intervention studies, such as core symptoms of Autism, adaptive functioning, IQ, and challenging behavior (6–9). The waxing and waning of target outcomes across development may contribute to the variable treatment response, but it is difficult to differentiate the sources of variability under the traditional experimental designs. For instance, some target treatment outcomes may decrease over a longer time span as individuals grow out of certain behaviors (e.g., from non-verbal to verbal communication), resulting in an artifact of reduced treatment response (10). Although the traditional RCT design has proven to be invaluable and thought of as the gold standard of evidence for the study of other—mostly physical—disorders, the derived findings are often limited in generalization beyond the trial sample given the restrictions mentioned above (11). Considering the idiosyncrasies of neurodevelopmental disorders where behavioral manifestations are often dynamic and heterogeneous in nature, there is a need to rethink how we approach the study of Autism intervention ingredients, including both predictors and outcomes, to better elucidate *what* works for *whom, why*, and *when*.

## Capturing Individual-Level Variability in Treatment Outcomes

**Figure 1:**
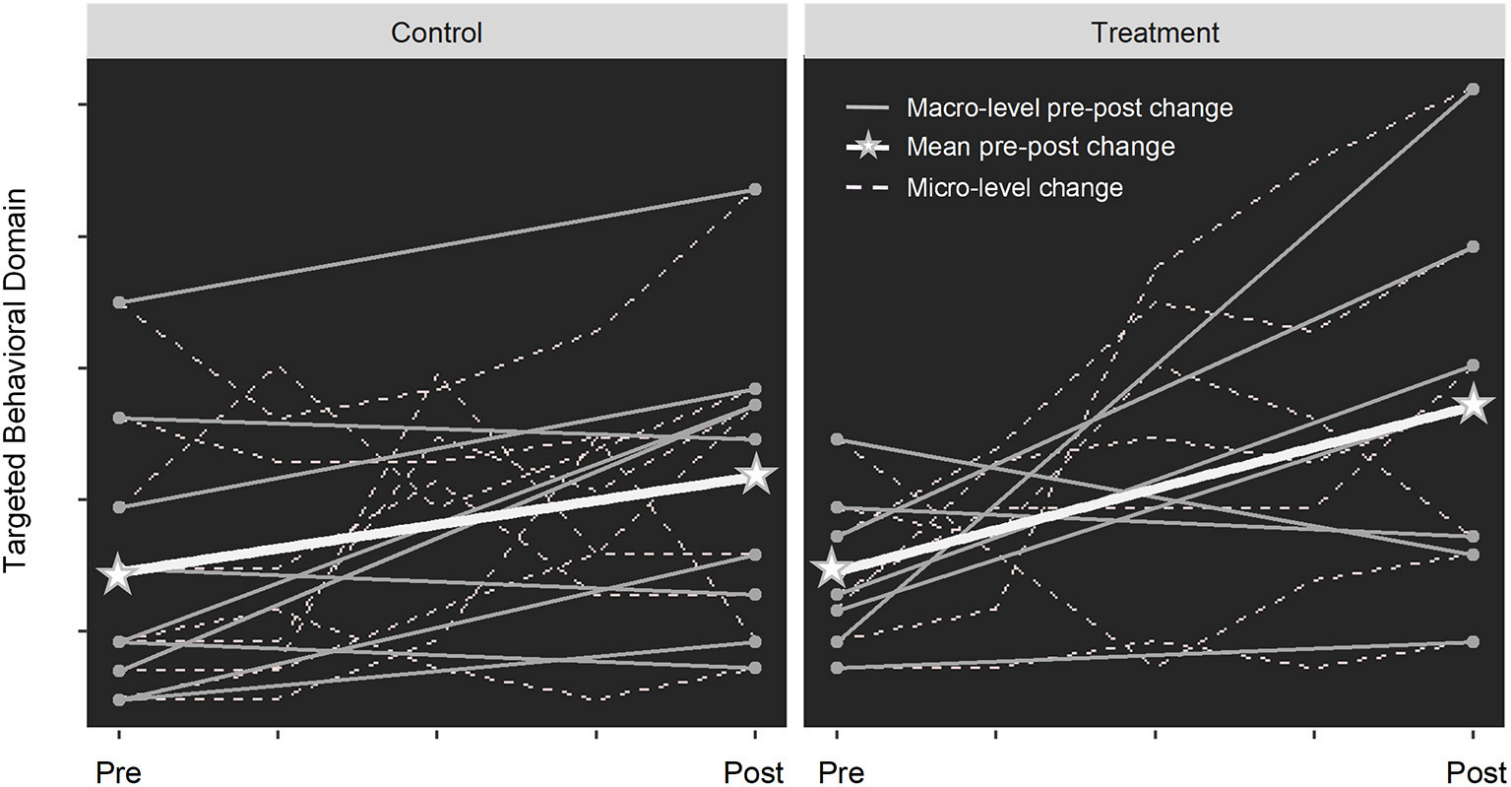
The “black box” of treatment effect in RCTs. In an RCT design, participants are randomized to treatment or control groups with matched baseline characteristics. When only calculating the average change from pre- to post-treatment (thick white lines), the larger increase observed in the treatment group may lead to the conclusion that the treatment is effective (assuming significant group difference), despite the individual-level heterogeneous response (gray lines). When breaking the treatment period into smaller intervals, the micro-level change (dashed lines) reveals that the rate of change varies across individuals over time, indicating time-varying treatment effects. Regarding “opening the black box”, we are not referring to unblinding the clinical trial procedures, but rather adopting study designs (e.g., more frequent data collection with more refined outcome measures over a longer time span) and analytical approaches (e.g., trajectory analyses) that allow for examining the finer-grained changes in treatment response.

In classical RCTs and quasi-experimental designs, it is common to collect outcome data at pre- and post-treatment, sometimes with post-treatment follow-up. While this satisfies the purpose of inferring whether the treatment is more effective than placebo, the “black box” of what happens *during* treatment remains unopened (see Figure 1). Further, the treatment-related change is often treated as a “chunk” averaged across individuals (e.g., average treatment effect, average effects on the treated) rather than a continuous process over time (e.g., within-person change) for each individual. While strategies such as subgroup analysis and propensity scores based on a priori groupings (e.g., sex) can be used for addressing heterogeneous treatment effects (12), it could still be problematic when observations do not correspond to individual experience or behavior in a non-ergodic (i.e., non-stationary and variable) behavioral change process in the real world, thus limiting the generalization and replication of the findings (13, 14). It also poses challenges in differentiating between individual treatment response and random variability that may bias the evaluation of treatment response. Some potential sources of bias include natural fluctuations of treatment outcomes, response bias (e.g., tendency to report favorable outcomes), practice effects, statistical artifacts (e.g., regression to the mean, ceiling/floor effect) (15). Finally, although randomization helps to increase the internal validity of group comparisons by making the factors associated with unobserved uncertainty equitably distributed across the treatment and the control groups, meaningful individual variability might also be distributed across the two groups. When sample size is small and/or individual variability is not well addressed with appropriate analytical approaches (e.g., accounting for within-group variations), there could be a higher probability of type II errors and thus reduced power to detect effects (16).

Although recent advances in longitudinal designs and analyses for observational studies have contributed to a better understanding of the heterogeneity of progression of Autism-related phenotypes both *within* and *between* individuals and over time—i.e., chronogeneity (17, 18)—how treatment outcomes are approached in Autism intervention research remains limited in addressing individual-level variability with respect to time. To date, there are only a handful of larger-scale intervention studies that describe developmental trajectories of intervention outcomes using approaches accounting for both within-person and between-person differences, such as multilevel modeling and latent growth curve analysis. In an RCT study (10), variable trajectories of joint attention behaviors were observed among a group of preschool-aged children diagnosed with Autism over the course of social communication intervention and 5-year follow-up, where the change patterns were associated with treatment assignment and diagnostic status at the exit. A recent observational study (19) reported an overall increase across diverse language trajectories between the entry and exit of an early intensive behavioral intervention (EIBI) program among preschool-aged children with Autism, with steeper improvements predicted by younger age, higher cognitive abilities, and lower symptom severity at baseline. Another observational study (20) examined the growth curve of autistic children's developmental outcomes across several time-points during applied behavior analysis (ABA) intervention and found that symptom severity, primary language spoken at home, and child's sex, but not treatment intensity and age of entry, were significant predictors of growth rates in certain outcomes during the intervention. Along with another observational EIBI study for children with Autism (21), different rates of improvement in treatment outcomes were observed across time-points during the period of intervention. For instance, many children tended to show patterns of exponential negative growth (faster improvement in the beginning followed by decelerated progress after). These findings suggest that treatment-related change is developmentally variable, and differences in baseline characteristics within treatment groups can be associated with various treatment responses or lead to different treatment outcomes. Also, the rate of change may vary during and beyond the intervention, potentially in a non-linear trend that is often hard to observe with limited data points. This indicates that there may be a time window for certain groups of people to better respond to the treatment.

Despite emerging evidence on the complex patterns of treatment-related change, more research that captures fine-grained variability over time is needed for informing personalized intervention in Autism. The fundamental issue might lie in the imbalance between simplicity and complexity when approaching treatment-related change with a lack of respect to the role of time in the risk and resilience process (22), thus limiting the field from yielding robust, meaningful, and translatable findings. Below we discuss some empirical gaps, methodological challenges, and opportunities that could be drawn from other fields, as well as an illustrative example for autism researchers to plan for “next steps”.

### Research Questions/Hypotheses

While Autism intervention science often poses the question of *what* works for *whom*, and *why*/*when* the change happened, the main research question tested empirically is *whether* the change happened due to the specific treatment. Although the latter question is foundational for demonstrating the effectiveness of treatment, it may not be sufficient in providing generalizable information on applications outside the clinical-trial settings where more variability related to individual differences or time is expected. Aside from confirming *whether* the treatment works, we may also explore the overall *shape* (i.e., progression) of change in specific proximal or distal treatment outcomes, *when* the greatest amount of change or inconsistent rate of change occurs, and *how* these patterns of change are associated with certain individual characteristics. Answers to these questions would inform the development of more tailored treatment programs that are more targeted and better timed for optimal response.

### Design/Measurement

These types of research questions highlight the need for refining the tracking of treatment outcomes through appropriate study designs, including:

- More frequent measurement occasions at shorter timescales to capture finer-grained behavioral change processes of treatment targets: While intensive data collection of proximal treatment targets is a common practice in EIBI, individual variability in behavioral change has rarely been addressed. Recently, intensive longitudinal (IL) methods, such as ecological momentary assessment, experience sampling, and daily diary, have been increasingly adopted in the field of psychopathology to better capture the temporal dynamics of symptoms and functions, thus allowing for better elucidation of treatment effects mechanisms (23, 24). While it remains challenging to collect longitudinal data with validated tools in behavioral research, the recent advance in remote monitoring and telehealth methods as well as the use of accelerated longitudinal design could facilitate the feasibility of more intensive behavioral data collection during clinical trials (24, 25).
- More refined and psychometrically validated behavioral constructs as treatment outcome measures: Given the multidimensional clinical features of Autism and associated challenges, it would be useful to have measures that capture *specific* domains (or sub-domains) of targeted outcomes and other key neurobehavioural constructs (e.g., specific joint attention skills instead of a general social communication composite score) at multiple time points to be able to examine the interplay of different treatment outcome domains over time, as well as to better account for the heterotypic development (i.e., age-dependent behavioral manifestations) of outcomes during long-term follow-up.

### Analysis

Future Autism intervention research may benefit from applying the learnings achieved in observational studies describing heterogeneous developmental trajectories. Specifically, analytical approaches for studying *between-person* differences in *within-person* change (e.g., latent growth modeling and multilevel modeling), and person-centered approaches for identifying homogeneous subgroups (e.g., growth mixture modeling), can contribute to better capturing individual treatment-related change over time. These approaches allow for addressing a variety of development-related complexities, such as non-linear trajectories, time-varying predictors, and interactions across multiple treatment outcome domains. They are also flexible in dealing with some common challenges in intervention studies, such as missing data and non-normally distributed measures (26). It should be noted that these approaches often require at least three time-points of panel data to estimate linear latent trajectories or linear random effects and four time-points for capturing non-linear within-person and between-person changes (27).

### Illustrative Example

te Brinke et al. (28) recruited a total of 108 adolescents with elevated externalizing behavior, who were randomized to either a treatment (emotion regulation training) group or a control group. Emotion regulation strategies and externalizing problems were assessed at baseline and at two treatment phases. At each phase (spanning 3–7 weeks), self-reports of emotion regulation difficulties and aggression were collected weekly via smartphones. Aside from examining the group differences in distal treatment outcomes (i.e., emotion regulation strategies and externalizing problems), this design allowed to examine the effect of treatment manipulation (alternating the sequence of cognitive or behavioral approaches) on proximal outcomes (i.e., emotion regulation difficulties and aggression) by modeling their piecewise trajectories across individuals in the treatment group. Similarly, autism researchers may apply such design to examine the trajectories of distal treatment outcomes (e.g., expressive language) between groups as well as finer-grained with-person changes in proximal treatment outcomes (e.g., specific joint attention skills) through more intensive data collection. Such an approach would also allow for examining the potential effect of changing intervention ingredients (e.g., sequence and dosage), which would be particularly useful under an adaptive intervention design.

## Modeling Dynamic Prediction of Treatment Response

**Figure 2:**
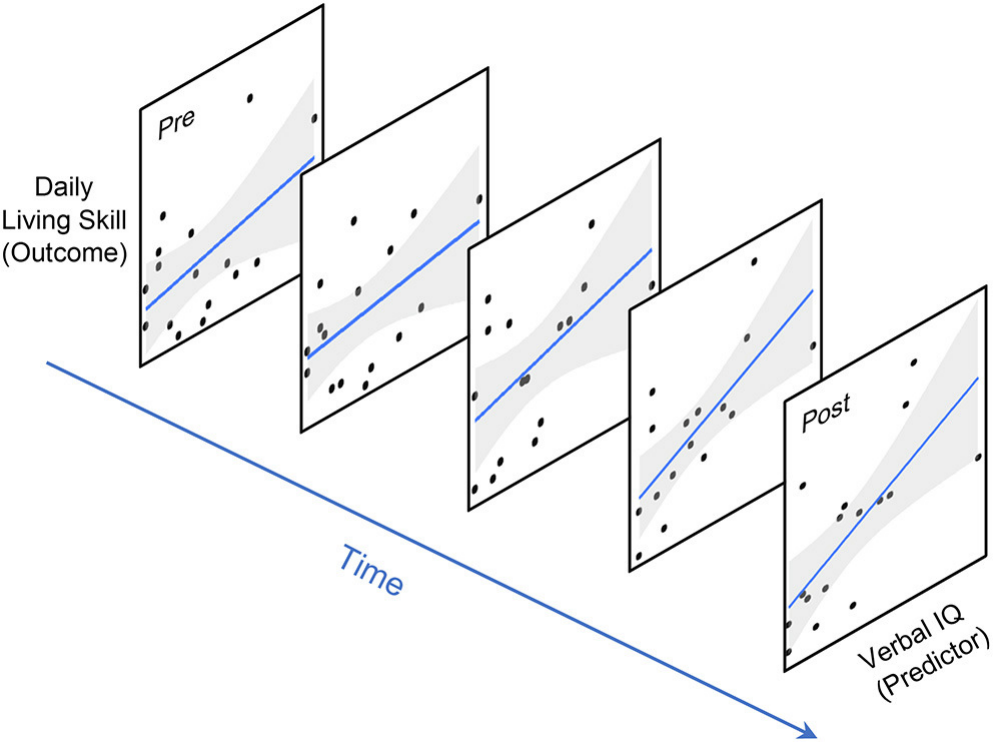
Time-varying prediction of treatment outcome. Conventional linear regression approaches assume the effect of predictors/covariates (e.g., IQ) on treatment outcomes (e.g., adaptive skills) to be static or constant over time. In contrast, a time-varying prediction model estimates their association as a function of time.

As we continue to advance our work on describing treatment-related change over time, it is also important to identify predictors of “more responsive” trajectories (e.g., higher rates of improvement, longer maintenance of treatment effects). As reported in the intervention studies mentioned above (19, 20), some child demographics (e.g., age, sex) and characteristics at baseline (e.g., IQ, level of symptoms) were associated with different trajectories during and/or after the intervention. What remains unclear, however, is the dynamic processes between predictors and treatment outcomes underlying these variable trajectories. Conventionally, predictors and their effects are treated as “static” based on the assumptions that, for example, cognitive and language skills do not change beyond the baseline and their effects on intervention outcomes hold constant over time. However, as demonstrated by many longitudinal studies, the outcomes and predictors of interest are often not static [e.g., IQ and symptoms of Autism; (8)] and may have dynamic associations with each other over the period of observation [e.g., language and social skills; (29)] among individuals with Autism. Moreover, major life changes, such as a transition to school and the COVID-19 pandemic, may “disrupt” children's trajectory outcomes and their associations with predictors (30, 31). Changing intervention components (e.g., types, dose, duration, intensity of treatment) may also influence such dynamics. Recently, adaptive intervention approaches (e.g., sequential multiple-assignment randomized trials, SMARTs) have represented a promising strategy to personalize Autism intervention (32), where participants are randomized into different sequences of intervention options according to their response to treatment. These “smarter” intervention approaches require “smarter” analytic methods to better address treatment-related change. And even in the case of predictors that are invariant in nature (e.g., sex), the magnitude of their predictive effect may still vary across the course of intervention and/or development [e.g., interactions between sex and age for comorbid symptoms in children with Autism; (33)]. All these complexities regarding the prediction of treatment-related change point to the need for more “time-sensitive” approaches, such as dynamic prediction modeling that allows for examining time-varying effects on treatment outcomes (see Figure 2).

The concept of *dynamic prediction* is not new to the field of psychopathology, which has been adopted in intervention studies for alcohol or drug addiction and affective disorders [e.g., (34, 35)] as well as in non-intervention studies such as the prediction of mental disorder onset and progression (36, 37). The idea behind the dynamic prediction is to approach psychopathology as a system rather than as a category (37) through capturing the reciprocal relation between trajectories of interest (e.g., treatment outcomes) and their etiologically and clinically relevant time-varying predictors (34). Recently, as a response to the impact of COVID-19, a dynamic clinical prediction model has been proposed to adapt to the constantly evolving healthcare system, where predictors as associated with changes in population demographics, prevalence of disease, and clinical practice paradigms are taken into account for decision-making (38, 39). From an analytic perspective, changes that arise over time (beyond experimental control) may introduce uncertainty to prediction models and result in “calibration drift” (i.e., less accurate predictive ability over time) (40). Thus, establishing prediction models with only baseline predictors may under-utilize the available information and thus limit predictive ability and replicability (41). While our current knowledge about predictors of treatment outcomes in Autism remains inconclusive due to several conceptual and methodological limitations, such as a lack of theory-driven models with attention to individual differences (42), the missing piece of “time” may be a major factor underpowering the detection of meaningful effects.

The concept of dynamic prediction also applies to the study of treatment mediation given the nature of mediators as outcome predictors. Mediation is commonly studied with regression-based approaches in intervention studies to understand treatment processes and mechanisms. Such approaches often assume mediation effects to be linear and thus ignore that independent variable, outcome variable and mediator are typically not in a strictly unidirectional and static relation, but instead in a bidirectional relation that may change over time (42). As demonstrated in a large-scale RCT study with long-term follow-up for a parent-mediated social communication intervention targeting children with Autism (43), while the treatment effect on parental synchrony (mediator) attenuated over time, the treatment effect on child outcomes did sustain at follow-up, indicating that the mediation mechanism could vary across different stages of intervention. Such finding also supports the theoretical foundation behind development-based intervention approaches (e.g., naturalistic developmental behavioral interventions), in which developmentally-appropriate precursor skills are targeted to improve developmental outcomes. Thus, a move from static to dynamic approaches of examining predictive effects, including moderation and mediation, would not only facilitate our understanding of why and when treatment response becomes differential across individuals, but also better reflect the rationale of developmentally grounded intervention approaches.

Given the advances across the broader field of psychopathology in addressing the dynamic nature of the predictor-outcome relation, as contrasted with the common practice of studying this interplay as static in Autism intervention science, we suggest that future research may want to identify time-varying predictors or covariates of treatment outcomes based on theory and existing evidence. Here we raise some potential challenges and opportunities regarding dynamic prediction of treatment outcomes, along with an illustrative example that might be applied to Autism intervention research.

### Research Questions/Hypotheses

As discussed above, some common baseline predictors, such as cognitive and language skills, may change over time and have a dynamic relation with each other and with treatment outcomes. Mediating effects could also vary across time, such as the effect of parent responsiveness on child's treatment outcomes during parent-mediated intervention vs. follow-up. In this regard, some examples of “time-sensitive” research questions that can be asked include: (1) When (e.g., 1 month upon entry, 6 months after exit) does the treatment effect become active or reduced? (2) How do individuals with certain characteristics or in different contexts (e.g., verbal vs. non-verbal, various intervention elements, levels of environmental support) differentially respond to the treatment over time (i.e., time-varying moderation of treatment outcome)? (3) Does the mediating effect on treatment outcome (e.g., parental responsiveness on child's social response) vary over time? (4) When do two or multiple behavioral domains of interest (e.g., core symptoms and comorbidities) become “decoupled” as the result of the intervention? These “time-sensitive” research questions could yield findings that fill the empirical gaps in the Autism intervention research regarding treatment timing and underlying mechanisms.

### Design/Measurement

Modeling dynamic prediction of treatment response requires repeated data collections of (lagged or concurrent) outcome and predictor variables with adequate coverage across the time span. And as with any longitudinal analysis, the assumption of measurement invariance across time should be met. We note that some challenges which have hampered Autism intervention research historically, such as the burden of repeated measurements on both participants and assessors, low recruitment numbers and high attrition rates (that lead to smaller sample sizes), are still relevant here. However, lessons and opportunities could be drawn from cross-site collaborations and consortiums for genomic and biomarker data that have been developed over the past decades in the field of Autism research (44, 45) for increasing sample sizes as well as enhancing data sharing and harmonization of clinical trial data, which would allow researchers to address more complex but relevant research questions. The Autism research community needs to work together to make it possible to identify meaningful predictors of treatment response through precision approaches (3).

### Analysis

As a direct extension from the widely adopted cross-lagged panel models in longitudinal studies, the incorporation of time-varying covariates could be achieved by specifying random intercept factors that represent the person-specific deviations from mean trajectories at a specific time-point (46). A similar idea can be also applied to multivariate latent curve modeling with structured residuals that capture time-specific within-person differences in the association of multiple trajectories (e.g., the association between trajectories of treatment outcome and predictor) (47). Other novel methods which have been increasingly used in the field of psychopathology include time-varying effect modeling [TVEM; (48)], dynamic structural equation modeling [DSEM; (49)], and joint modeling (50). A shared characteristic of these methods is the non-parametric or semi-parametric estimation of regression coefficients for time-series data without a priori constraints on underlying trajectories and shapes of coefficient functions. These methods have been applied to intervention and prevention for addiction and affective disorders [e.g., (51–53)], as well as detection of transition to psychosis [e.g., (54)], and thus may be useful candidates to be applied and tested in Autism intervention science. Survival analytical approaches that are widely adopted in medical and epidemiological research, such as Cox proportional-hazards regression models, which assume log-linearity in covariates, could also be used to examine the time-varying effects of covariates (55). Finally, Bayesian approaches could be applied to handle time-varying coefficient models with greater complexity (e.g., multiple random effects) (56).

### Illustrative Example

Wright et al. (51) examined the impact of co-occurring anxiety on depression treatment outcomes among 78 outpatients over the course of psychotherapy. The patients received either traditional psychotherapy for depression or a variant that also targets anxiety. Depression and anxiety symptoms were assessed by clinicians at each of the 16 weekly treatment sessions. Instead of using baseline anxiety as a predictor, the researchers examined the dynamic associations (i.e., time-specific coefficients) between anxiety and depression using TVEM to clarify whether anxiety and depression “decoupled” as treatment proceeds. They also examined whether the time-varying associations differ between groups who received different versions of treatment at certain time-points. Autism researchers could also apply a similar approach to explore research questions related to co-occurring symptoms (e.g., repetitive and restricted behaviors and anxiety) during behavioral interventions, parent-child dyads (e.g., parent responsiveness and child's joint attention skills) during parent-mediated interventions, or inclusion of predictors that may change in nature (e.g., cognitive and language skills). This would allow for elucidating how and when the covariates of interest contribute to treatment targets or interact with intervention elements at an individual level.

## Conclusion

We discussed here two important empirical gaps in Autism intervention science that, to date, has been relying on observational or experimental designs (including RCTs) predominantly characterized by: (1) evaluating treatment effects based on group comparisons of mean pre-post changes in general outcome domains; and (2) studying prediction of treatment outcomes as a static phenomenon. The failure to measure more real-time treatment response, particularly under a pre-post design, may lead to a biased inference of treatment effects. Moreover, while it is reasonable to “control for” static predictors of intervention outcomes, the findings should be cautiously interpreted given the untested assumption that the predictors pose effects on the target outcomes that do *not* vary in strength over time. This, however, may undermine the predictive accuracy of treatment response and limit the generalization of findings to autistic populations with heterogeneous developmental profiles in real-world contexts. Given the dynamic and developmental nature of psychopathology (57), such as the gene-environment interplay that may impact developmental outcomes in Autism (58, 59), it is important to take temporal and contextual dimensions of treatment effects into account to elucidate *why* and *when* the intervention works or does not work. We discussed several methodological challenges and potential solutions for addressing these empirical gaps when designing Autism intervention studies. Adopting a dynamic lens can help researchers and clinicians to better understand the adaptive developmental processes to positive or negative changes associated with intervention or environment (59), whose importance is underscored by the pandemic's significant impacts on autistic individuals and their families (31, 60). As the field is entering the era of stepped and personalized healthcare (61), there is a need to pause, rethink, and discuss an intervention research agenda that better addresses the developmental and dynamic nature of Autism, and to adopt methodological approaches that support the shift of focus from *macro* to *micro*-level change, as well as from *static* to *dynamic* prediction of change. Such a paradigm shift would contribute to the refinement of personalized interventions tailored to heterogeneity across development (i.e., chronogeneity) so that interventions and services could be delivered to autistic individuals and their families in a timely, targeted, and adaptive manner.

## Data Availability Statement

The original contributions presented in the study are included in the article/supplementary material, further inquiries can be directed to the corresponding author/s.

## Author Contributions

SG and Y-JC contributed to the conception of this manuscript. Y-JC drafted the manuscript with comments and edits from ED and SG. The final version of the manuscript was read and approved by all the authors.

## Funding

This work was supported by the McMaster Children's Hospital Chair in Autism and Neurodevelopment (to SG) and the Offord Center for Child Studies postdoctoral fellowship (to Y-JC).

## Conflict of Interest

The authors declare that the research was conducted in the absence of any commercial or financial relationships that could be construed as a potential conflict of interest.

## Publisher's Note

All claims expressed in this article are solely those of the authors and do not necessarily represent those of their affiliated organizations, or those of the publisher, the editors and the reviewers. Any product that may be evaluated in this article, or claim that may be made by its manufacturer, is not guaranteed or endorsed by the publisher.
